# Structure–activity correlation of thermally activated graphite electrodes for vanadium flow batteries†

**DOI:** 10.1039/d2ra02368g

**Published:** 2022-05-11

**Authors:** Adrian Lindner, Hannes Radinger, Frieder Scheiba, Helmut Ehrenberg

**Affiliations:** Institute for Applied Materials, Karlsruhe Institute of Technology 76344 Eggenstein-Leopoldshafen Germany hannes.radinger@kit.edu

## Abstract

Thermal activation of graphite felts has proven to be a valuable technique for electrodes in vanadium flow batteries to improve their sluggish reaction kinetics. In the underlying work, a novel approach is presented to describe the morphological, microstructural, and chemical changes that occur as a result of the activation process. All surface properties were monitored at different stages of thermal activation and correlated with the electrocatalytic activity. The subsequently developed model consists of a combined ablation and damaging process observed by Raman spectroscopy, X-ray photoelectron spectroscopy and scanning electron microscopy. Initially, the outermost layer of adventitious carbon is removed and sp^2^ layers of graphite are damaged in the oxidative atmosphere, which enhances the electrocatalytic activity by introducing small pores with sharp edges. In later stages, the concentration of reaction sites does not increase further, but the defect geometry changes significantly, leading to lower activity. This new perspective on thermal activation allows several correlations between structural and functional properties of graphite for the vanadium redox couple, describing the importance of structural defects over surface chemistry.

## Abstract 2

Structural changes on the surface of graphite felts after thermal activation were monitored. Fundamental correlations led to a new model to explain the morphological evolution and its effects on the electrocatalytic activity in vanadium flow batteries.

## Graphical abstract

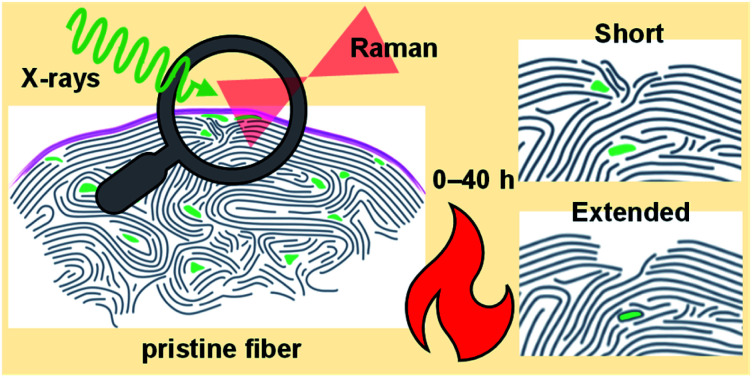

## 1. Introduction

With the growth in renewable energy generation, the need for sustainable and lossless long-term storage is rising. The vanadium flow battery (VFB) is a promising candidate due to its large scalability, low energy loss, and long lifetime.^1^ While both metals and metal oxides are being investigated as electrocatalysts, carbonaceous species such as graphite felt (GF) are the predominant choice for electrode materials.^2,3^ However, challenges arise from the slow reaction kinetics of GF, leading to many efforts in the exploration of suitable modification processes to improve the electrocatalytic activity towards the vanadium redox couples.^4^ Thermal oxidation is widely used on an industrial scale due to its effectiveness and simplicity. Nevertheless, our understanding of the relevant structural changes that occur at the surface of GF is very limited. Not only one property is altered at a time, but microstructure, surface chemistry, and morphology change simultaneously. As a result, conclusions in literature diverge regarding the parameters thought to be responsible for the electrocatalytic activity.^5^ Using other oxidative processes such as chemical and electrochemical activation, similar uncertainties exist in explaining and interpreting the results.^6,7^

Thermal treatment of GF at 400 °C for 30 h was already proposed in the early 1990s, and the parameters have not changed significantly over the years.^8^ The higher cell efficiencies were initially attributed to an increased concentration of surface oxygen. Two influencing factors have been the subject of further investigation: treatment time and activation temperature. The use of shorter treatment times at higher temperatures such as 500 °C for 4 h appeared to have similar effects on GF, leading to an increase in active surface area and cell efficiency.^9^ A reduction in overvoltages was observed after treatment in an oxygen-enriched atmosphere, whereas activation without oxygen in the atmosphere showed no significant improvements.^10^ While these conclusions are still relevant, other interpretations have since been made. It has been suggested that an increase in surface area and roughness, due to damaging oxidation of GF, is responsible for the increased activity instead of chemical surface functionalization.^9,11,12^

X-ray photoelectron spectroscopy (XPS) is used to study the chemical composition of an electrode surface. In addition to quantifying the type and concentration of oxygen groups, it is possible to distinguish between sp^2^ and sp^3^ hybridized carbon. It has been shown that both, differently bound oxygen and carbon hybridization, affect the reaction kinetics.^13,14^ Surface oxygen, while most probably not an active reaction site, increases the polarity for better electrode wettability and the surface area accessible to the electrolyte.^15^ Raman spectroscopy allows an in-depth study of the structural properties of carbon-based materials.^16–18^ It is possible to characterize not only the degree of disorder but also the type of defects.^19,20^ Studies on model electrodes have shown that edge sites allow faster electron transfer for vanadium redox reactions.^21^ For GF, it was subsequently demonstrated that defect density is related to electrocatalytic activity.^22–24^ Recent computational and experimental approaches suggest that oxidation can be considered as a by-product of lattice defects, suggesting that future studies should focus on non-oxidative activation techniques.^25,26^

A thorough understanding of how a modification technique alters the surface properties of an electrode can be considered key to a mechanistic understanding of chemical reactions. Finding correlations between the number of lattice defects, chemical composition, and electrochemical performance will enable this approach. Therefore, we have studied in detail the interplay between electrocatalytic activity and physiochemical properties for thermally activated GF. Scanning electron microscopy (SEM) and Raman spectroscopy were used to study defects at the micro- and nanoscale in relation to oxidation time. The chemical composition was monitored by XPS. A detailed comparison of all parameters allowed us to propose a model for thermal activation, and sheds light on the important properties to effectively use GF as an efficient electrochemical energy converter. Furthermore, we show how the above-mentioned analytical tools can be used in combination to develop a holistic view of the structural properties of graphite.

## 2. Experimental section

### 2.1. Electrode and electrolyte preparation

Pure graphite felt (Sigracell GFD 4.6, SGL Carbon) is cut and thoroughly washed in an ultrasonic bath with acetone and distilled water before being dried in a drying oven at 80 °C for 24 hours. The clean and dry GF is then transferred to an alumina crucible and thermally treated in a muffle furnace at 400 to 500 °C under ambient conditions with a heating rate of 3 K min^−1^. The treatment time varied from 4 to 40 h. The GF was then cooled to room temperature in the oven. The electrolyte for the positive half-cell was prepared by dissolving 0.1 M VOSO_4_ (Alfa Aesar) in 2 M H_2_SO_4_ (Emsure). The negative half-cell electrolyte was prepared by cycling the positive electrolyte in a full cell.

### 2.2. Physicochemical characterization

The mass loss of GF under artificial air atmosphere was monitored by thermogravimetric analysis (TGA) in a temperature range from 30 to 900 °C at a heating rate of 10 K min^−1^.

Raman spectroscopy was performed using a Horiba Scientific LabRAM HR Evolution equipped with a 633 nm laser and a 100× magnification objective, resulting in a spot size of ∼2 µm. High wavelength and low laser power are required for high resolution and to avoid material damage. Peak intensity ratios are used to determine various features, using Gauss–Lorentz profiles after spline background correction. *I*_D_/*I*_G_ and *I*_D_/*I*_D′_ ratios were used to evaluate defect density and features.

The morphology of the samples was studied by scanning electron microscopy (SEM, Merlin, Carl Zeiss) at an accelerating voltage of 5 kV and a probe current of 150 to 500 pA.

The chemical composition was analyzed by XPS using a K-alpha^+^ spectrometer (Thermo Fisher Scientific) with monochromatic Al-K_α_ radiation (*E*_photon_ = 1486.6 eV) and a spot size of ∼400 µm. Survey spectra were recorded with a pass energy of 200 eV, and detail spectra with 50 eV. A Shirley background correction was applied and individual species were deconvoluted by Gauss–Lorentzian peak profiles. The asymmetry of the sp^2^ hybridized carbon was evaluated with a tail mix of 90% and a tail exponent of 1. The positions of the residual components in the C 1s region were constrained to the position of the sp^2^ carbon, and the FWHM (full width at half maximum) values were constrained to sp^3^ carbon with a tolerance of ±0.1 eV for each. The O 1s region was deconvoluted by several peaks spaced 1 ± 0.1 eV apart, with the FWHM constrained by ±0.1 eV.

### 2.3. Electrochemical characterization

Electrochemical measurements were performed using a BioLogic VSP potentiostat. Before each measurement, an electrochemical cleanup step was applied to the sample at a potential range of 0 to 0.5 V (positive half-cell) and 0 to −0.3 V (negative half-cell) at 100 mV s^−1^. For all electrochemical measurements, a three-electrode setup was used with GF as the working electrode, pristine GF as the counter, and Ag/AgCl (3 M KOH) as the reference electrode. Argon gas was used to deaerate the electrolyte and prevent dissolution of atmospheric oxygen. The GF was immersed in the electrolyte and centrifuged before measurement to ensure adequate wettability.

Two different CV measurements were performed: first, in a non-faradaic potential window with increasing scan rate from 10 to 250 mV s^−1^ to calculate the electrochemical double layer capacitance (EDLC). Second, between 0.2 to 1.6 V for the positive, and −0.05 V to −0.85 V for the negative half-cell response with scan rates from 1 to 10 mV s^−1^. The CV curves were *iR*-corrected using electrochemical impedance spectroscopy (EIS) at the OCV. EIS was performed in a frequency range from 1 Hz to 100 kHz. At an applied potential of 0.9 V and −0.450 V, the charge transfer resistance (*R*_CT_) of each vanadium redox reaction was evaluated. The impedance spectra were deconvoluted using the RelaxIS 3 software (rhd instruments). All measurements were described by the same equivalent circuit, using a resistor *R* for the junction and a *RQ* element for the electrode–electrolyte interface.

## 3. Results and discussion

### 3.1. Physicochemical properties

To determine the most effective activation parameters, the thermal stability of untreated GF was investigated by TGA measurements (Fig. S1†). Below 200 °C, only small changes in relative mass were observed, which can be attributed to evaporation of residual water or decomposition of organic residues. After that, a plateau was reached up to 500 °C; above, the material decomposed rapidly. Since thermal activation usually occurs over longer periods of time, the stability of the material was studied at a fixed temperature of 400 and 500 °C over a longer period (Fig. S2†). At 500 °C, more than 80% of the original weight of GF is lost within the first 10 h, while it is stable at 400 °C, designating this temperature as the choice for further experiments.

**Fig. 1:**
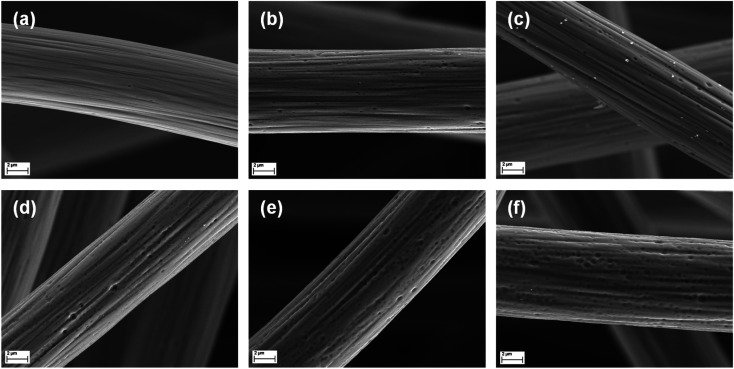
High-resolution SEM images of GF. A single fiber is displayed for (a) pristine and (b–f) thermally activated GF after (b) 8 h, (c) 16 h, (d) 24 h, (e) 32 h, and (f) 40 h at 400 °C.

The microstructural changes caused by the activation were visualized by SEM. Untreated GF (Fig. 1a) showed a smooth surface, except for the characteristic stripes parallel to the fiber length resulting from the fabrication process. Activation at 400 °C led to the formation of numerous small pore defects after 8 h (Fig. 1b). At longer heating times of 16 and 24 h (Fig. 1c and d), both the size and the number of defects present increased. After 32 h (Fig. 1e), the individual pores were still growing in size, but their edges appear less sharp than at shorter heating times. This trend became even more pronounced after 40 h (Fig. 1f), showing highly concentrated but dull defects.

**Fig. 2:**
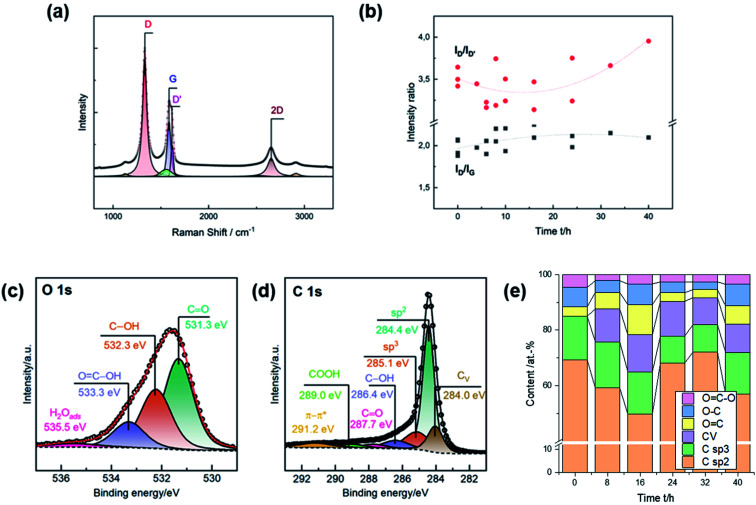
Physicochemical characterization of pristine and thermally activated GF. (a) Example of a deconvoluted Raman spectrum of GF; (b) intensity ratios of the D, G, and D′ bands. (c and d) XPS data of a selected GF, displaying all evaluated surface species in the O 1s and C 1s region; (e) quantification of the carbon–oxygen moieties.

Raman spectra were recorded for a more detailed characterization of the induced damages. The characteristic signals are the defect-induced bands D (∼1330 cm^−1^) and D′ (∼1620 cm^−1^), and the graphite-related band G at ∼1580 cm^−1^ (Fig. 2a). The *I*_D_/*I*_G_ ratio is typically used to determine the degree of disorder in carbonaceous material, while the *I*_D_/*I*_D′_ ratio provides information on the type of the defect. Due to the heterogeneity of GF, the activation was repeated several times to inspect multiple charges. Consistent with SEM observations, the number of defects increased during thermal activation up to 16 h, as evidenced by the increase in the ratio of D to G from ∼2.00 to ∼2.25 (Fig. 2b). Longer heating times did not lead to more disorder. The *I*_D_/*I*_D′_ ratio decreased from ∼3.5 for untreated GF, with a clear minimum between 8 h and 16 h. At longer durations, the ratio increased again. An intensity ratio of ∼3.5 is related to the presence of edge defects, consistent with the observation that the well-defined pores exhibit dull shapes after long activation times. Lower ratios originate from several edges being stacked in the direction perpendicular to the sheet.^27^

Oxygen concentration and sp^2^, sp^3^, and carbon vacancy (*C*_V_) content were assessed by XPS. The oxygen and carbon regions were deconvoluted to determine the content of individual surface groups (Fig. 2c and d). C=O (∼531.3 eV), C–OH (∼532.3 eV), and COOH (∼533.3 eV) groups were identified in the O 1s spectra. The fraction of graphitic (∼248.4 eV) and amorphous carbon (∼285.1 eV) was determined from the C 1s spectra. Ion bombardment of graphite in vacuum was in other studies used to associate another compound below sp^2^ carbon at ∼284 eV with carbon vacancies.^28^ In this work, all spectra were deconvoluted using this additional feature to investigate correlations to microstructural analysis by SEM and Raman spectroscopy.

All surface groups were quantified to study the evolution of the chemical composition as a function of the duration of thermal activation (Fig. 2e). The surface of untreated GF comprises about 69 at% sp^2^ carbon and 15 at% oxygen. In addition, sp^3^ carbon accounted for about 16 at%, and no vacancies were detected. The most noticeable change was an increase in *C*_V_ to ∼13 at% after 16 h of thermal treatment. Longer heating times of up to 40 h resulted in a subsequent decrease in vacancies to ∼10 at%. Similarly, the total oxygen content peaked after 16 h (∼22 at%). Further activation initially led to a decrease to ∼8 at% after 32 h, but then to a renewed increase to ∼18 at% after 40 h. The concentration of sp^3^ carbon showed no appreciable change during the first 16 h, but after 24 h there was a notable decrease and subsequent increase in sp^2^ carbon.

### 3.2. Electrochemical properties

**Fig. 3:**
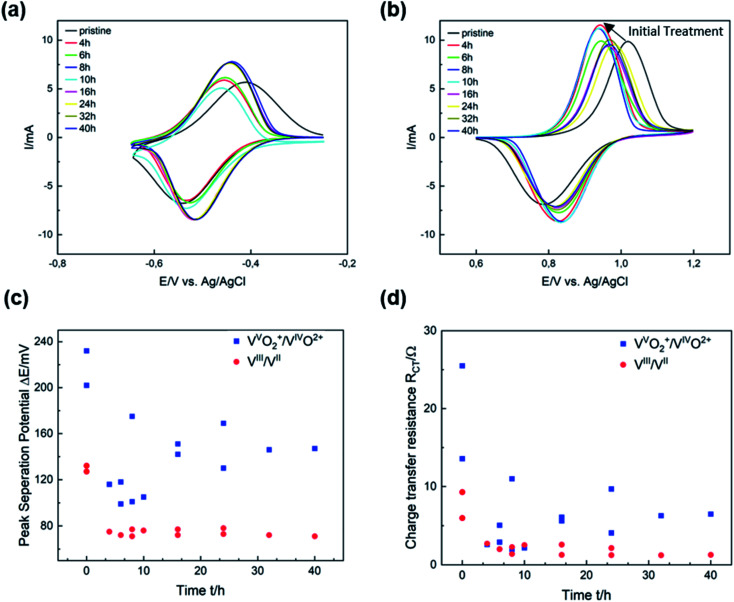
Electrocatalytic activity of thermally activated GF. (a and b) CV curves recorded in the (a) negative and (b) positive half-cell. (c) Peak separation potential in the negative and positive half-cell. (d) EIS at an applied potential in the negative and positive half-cell.

The electrocatalytic activity of thermally activated GF for the vanadium redox couples was evaluated by half-cell electrochemistry. CV curves were plotted for different activation durations and compared with untreated GF. Thermal activation significantly improves both the redox reversibility (*i.e.*, peak current ratio *i*_p_) and peak potential separation Δ*E*_p_ of the V^III^/V^II^ reaction (Fig. 3a). After 8 h, Δ*E*_p_ decreased from 130 mV for GF to 70 mV, while the *i*_p_ ratio increased from 0.84 to 0.91, clearly showing higher electrocatalytic properties of the treated GF. With prolonged activation, however, the potential separation grew and the peak currents shrunk, but high activity is restored after 24 h of thermal activation. For the V^V^O_2_^+^/V^IV^O^2+^ redox reaction, shorter activation times of 4 to 10 h resulted in a sharp decrease in Δ*E*_p_ from 230 to 100 mV as well as higher *i*_p_ ratios up to 0.78. Longer treatment durations of more than 16 h were detrimental to the activity and increased Δ*E*_p_ again to 150 mV.

In Fig. 3c, Δ*E*_p_ is plotted *versus* time to show that the catalytic activity for the V^III^/V^II^ redox reaction is almost unchanged after the first few hours of thermal activation. A similar trend was observed in the evaluation of charge transfer resistances (*R*_CT_) by EIS (Fig. 3d). For the V^III^/V^II^ reaction, the *R*_CT_ decreased from 6 Ω for GF to about 1 Ω after more than 24 h (Fig. S3†). Considering the V^V^O_2_^+^/V^IV^O^2+^ redox couple, the high *R*_CT_ of GF (25 Ω) was reduced to about 5.5 Ω after 16 h (Fig. S4†). The results obtained from CV and EIS show a different evolving behavior over the thermal activation period depending on the half-cell reaction. A more pronounced evolution over time is observed for the positive half-cell reaction. The two activity parameters discussed (Δ*E*_p_ and *R*_CT_) both show a dramatic decrease at low activation times between 4 and 12 h, followed by a subsequent increase. This corresponds to an improved electrocatalytic activity for short activation times, which is reversed by overlong heating. Over the entire period studied, both Δ*E*_p_ and *R*_CT_ are higher for the V^V^O_2_^+^/V^IV^O^2+^ than for the negative half-cell reaction. Moreover, the *i*_p_ ratios for the V^III^/V^II^ redox pair are closer to ideal reversibility (=1) over the entire temperature range, while for the positive half-cell the reversibility improves in the early stages of thermal activation but decreases again after more than 16 h (Fig. S5†).

### 3.3. Model for thermal activation

The results of microstructural characterization and surface chemical composition analysis revealed that thermal activation cannot be considered as a linear process. Instead, it showed a significant effect on the electrocatalytic properties within the first 8 to 16 h. This increase in activity corresponded to the formation of sharp edge defects, which were visualized by SEM and measured by Raman spectroscopy as an increase in the *I*_D_/*I*_G_ ratio. At the same time, an increased number of carbon vacancies was detected by XPS. Longer activation times resulted in decreased catalytic activity, which was accompanied by a change in defect geometry that exhibited large pores with blunt edges. In addition, the concentration of carbon vacancies decreased again. Furthermore, the changing oxygen content indicated that the OFGs were unstable at prolonged activation.

**Fig. 4:**
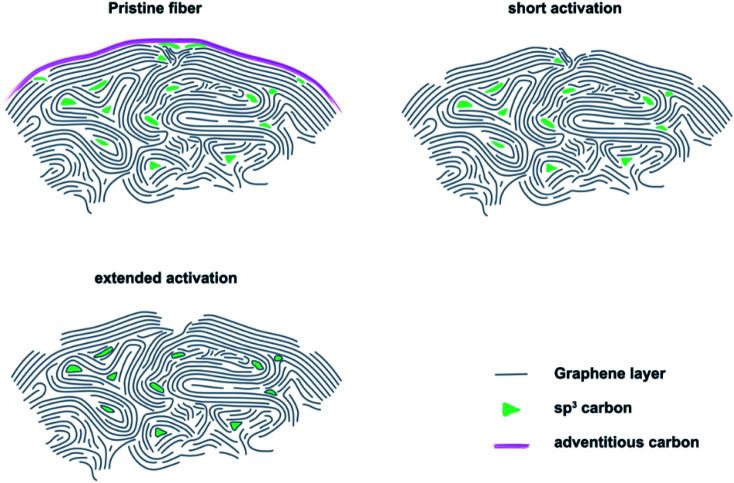
Schematic depiction of our three-stage model for the thermal activation of GF.

Based on our experimental observations, we propose a model for the thermal activation process that shows three important stages of GF (Fig. 4). On the outside, the felt electrodes consist of several, mostly sp^2^ hybridized graphite layers. The outermost layer is covered by adventitious carbon, which forms during prolonged atmospheric contact. When pristine GF is introduced into an oxidizing atmosphere, the adventitious carbon is removed and the outermost layer of intact graphite is damaged, leading to the formation of defects that are saturated by oxygen-containing groups. The defects formed in the initial phase of activation are small and have sharp edges, as determined by SEM and Raman spectroscopy. With increasing treatment time, they initially grow due to oxidation of sp^3^ hybridized carbon, which was quantified by XPS. However, the defect size is finite due to the slower oxidation of sp^2^ carbon under the applied conditions. This means that after an activation time of about 24 to 32 h, most defects have reached their final shape, which is confirmed by the electrochemical data showing no changes in *R*_CT_ after 24 h of treatment.

We conjecture the following mechanism for the thermal activation of GF: initially, small and sharp defects are formed at sites of structural weakness due to a higher local concentration of sp^3^ carbon. With increasing treatment time, more and more sp^3^ carbon is oxidized, leading to the growth of defects and a morphological change from sharp to blunt edges. As a result, the concentration of the active reaction sites declines, leading to increased *R*_CT_ and overall lower performance of the positive half-cell reaction. When the defects reach a certain critical size, all locally available weaker sp^3^ carbon is oxidized and defect growth stops. Only a higher temperature or other changes in atmospheric conditions could further increase the defect size at this point.

### 3.4. Structure–activity correlation

**Fig. 5:**
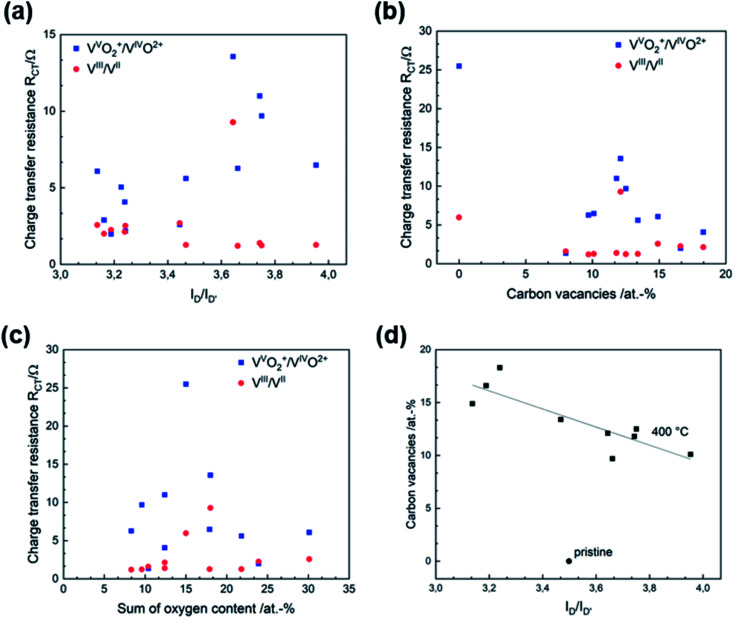
Structure–activity correlation of thermally activated GF. Correlation between (a) *R*_CT_ and defect type, (b) *R*_CT_ and *C*_V_, (c) *R*_CT_ and oxygen concentration, (d) *C*_V_ and defect type.

After developing a model describing the structural evolution of GF during thermal activation, correlations between physicochemical properties and catalytic activity can be established. Both *R*_CT_ and Δ*E*_p_ show a correlation with the geometry of the defects described by the *I*_D_/*I*_D′_ ratio (Fig. 5a and S6†). An increased *I*_D_/*I*_D′_ ratio resulted in decreased activity for the V^V^O_2_^+^/V^IV^O^2+^ reaction. In contrast, a slight improvement was observed for the negative half-cell. Looking at the SEM images, the loss of sharp pore edges after 16 to 24 h correlates well with the changing ratio in the Raman spectra. An *I*_D_/*I*_D′_ ratio between 3.0 and 3.5 was associated with edge-related defects. Longer activation times resulted in fewer edge defects, as evidenced by increasing *I*_D_/*I*_D′_ values above 3.5. Another correlation was observed between catalytic activity and the concentration of carbon vacancies determined by XPS (Fig. 5b). In both half-cell reactions, a higher number of vacancies resulted in higher activity.

According to several studies on the activity of carbon-based electrodes for VFBs, the oxygen concentration at the surface increases the electrochemical performance, especially for the negative half-cell. The underlying work does not allow a conclusive statement on the functionality of oxygen due to lack of correlations. Recent studies suggest that the functional groups of oxygen are overestimated, and most activation processes that generate oxygen at surface defects are overestimated for the charge transfer process, since activation procedures that create oxygen functional groups also increase other kind of defects like carbon vacancies.^5^Fig. 5c and S7† show the *R*_CT_ and Δ*E*_p_ in relation to surface oxygen content. Neither is the V^III^/V^II^ reaction affected by the number of oxygen groups, nor can a correlation between the activity of for the positive half-cell reaction and the presence of oxygen groups be established.

This complements our previous studies on that topic and once again showcases that oxygen has no positive effect on the catalytic activity of GF.^23,26^ We advocate a new approach to the concept of active reaction sites on graphite for vanadium redox reactions: instead of considering oxygen groups on the surface as active species, more efforts should be made to understand the quality and type of edge sites and carbon vacancies. According to our results, the positive half-cell reaction is the bottleneck when thermal treatment is used as an activation technique. Both the overvoltage and cell resistance were significantly higher. The ability of GF to catalyze the V^V^O_2_^+^/V^IV^O^2+^ redox couple depended strongly on the microstructural properties of the electrode. In comparison, the activity for the negative half-cell was largely unaffected by changes in microstructure and chemical composition.

Because of the very different effects of thermal activation on the activity of the vanadium redox reactions, the measurements were performed in half-cell configuration. Experiments in a full-cell introduce a new set of performance-limiting factors that make it impossible to distinguish between the influences, as shown in this work. The goal of this work was not to determine the best performing electrode, but to clarify the fundamental effects of thermal treatment.

In addition, SEM, Raman spectroscopy and XPS can be used complementarily to analyze the structural properties of graphite. These analytical tools evaluate the integrity of the carbon lattice and provide multiple insights into the defect composition from the micro to the nanoscale. The electrochemical activity correlated very well with the visual appearance of the pores in the SEM images, the *I*_D_/*I*_G_ and *I*_D_/*I*_D′_ ratio determined by Raman spectroscopy, and the *C*_V_ concentration in XPS. Fig. 5d shows how the *C*_V_ content and the *I*_D_/*I*_D′_ ratio are related. For thermally activated GF, a higher number of vacancies corresponded to a lower D to D′ ratio.

## 4. Conclusions

The interaction between microstructure, chemical composition and electrocatalytic activity of graphite felt electrodes has been studied. This work shows that defects in the graphite lattice effectively increase the activity for the redox reactions that take place in VFBs. The formation of these defects on the surface of GF occurs mainly in the early stages of thermal activation, with higher activation times leading to an increase in pore size and blunting of the edge geometry. SEM images and Raman spectra confirmed the time-dependent change in defect characteristics with a transition from sharp to dull edges. We propose a new three-step model which explains the structural and chemical changes on the surface of GF observed by typical analytical techniques such as Raman spectroscopy or XPS. Our model describes why research results correlating physicochemical properties and increased electrochemical activity as a result of thermal activation are sometimes contradictory. Moreover, the underlying results suggest that the V^V^O_2_^+^/V^IV^O^2+^ redox reaction is the kinetic bottleneck in VFBs. Both *R*_CT_ and Δ*E*_p_ are higher and more sensitive to surface structure at the same electrode. However, thermal activation is able to decrease Δ*E*_p_ by ∼50% and the *R*_CT_ by ∼80%. We have shown that the combination of SEM, Raman spectroscopy, and XPS is very powerful for an in-depth study of the defect structure of GF. New opportunities arise from the evaluation of the *I*_D_/*I*_D′_ ratio in Raman spectra and the proposed *C*_V_ signal below sp^2^ carbon in XPS to learn more about the nature and geometry of disorder.

## Conflicts of interest

There are no conflicts to declare.

## Supplementary Material

RA-012-D2RA02368G-s001
