# Supplementary material for: Structure–activity correlation of thermally activated graphite electrodes for vanadium flow batteries
Source: RSC Adv. 2022 May 11;12(22):14119–26. doi: 10.1039/d2ra02368g (PMC9092384; doi:10.1039/d2ra02368g)
Supplement: RA-012-D2RA02368G-s001 [file RA-012-D2RA02368G-s001.pdf]

Electronic Supplementary Material (ESI) for RSC Advances.
This journal is © The Royal Society of Chemistry 2022

Supporting Information

for

Structure–Activity Correlation of Thermally Activated Graphite Electrodes for Vanadium Flow Batteries

Adrian Lindner, Hannes Radinger*, Frieder Scheiba, Helmut Ehrenberg

Institute for Applied Materials, Karlsruhe Institute of Technology, 76344 Eggenstein-Leopoldshafen, Germany

**hannes.radinger@kit.edu*

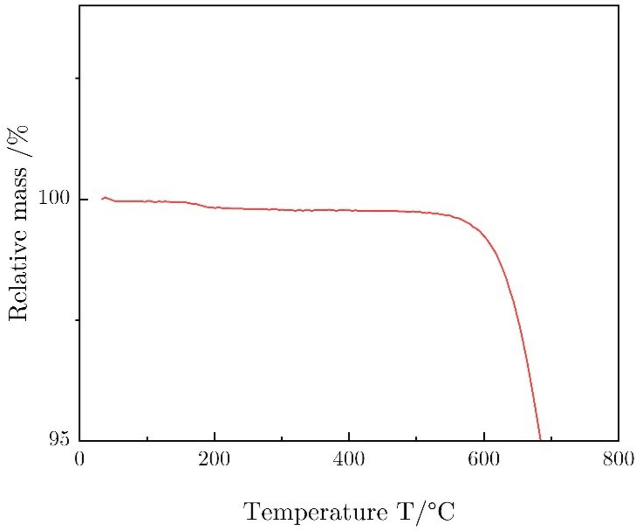

Figure S1. TGA measurement of pristine GF.

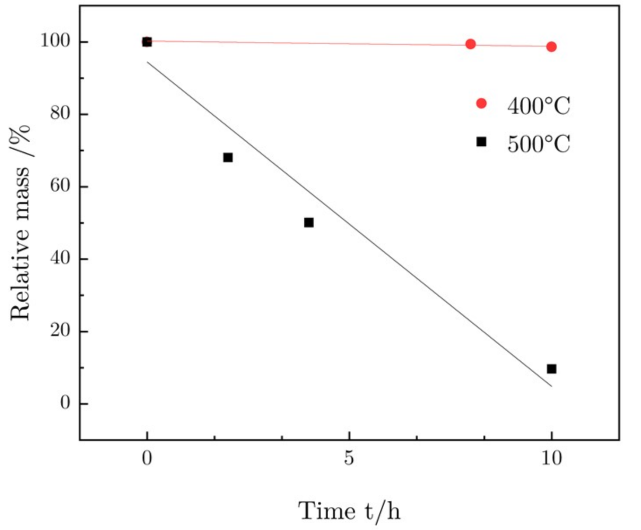

Figure S2. Mass loss of GF, determined by TGA measurements.

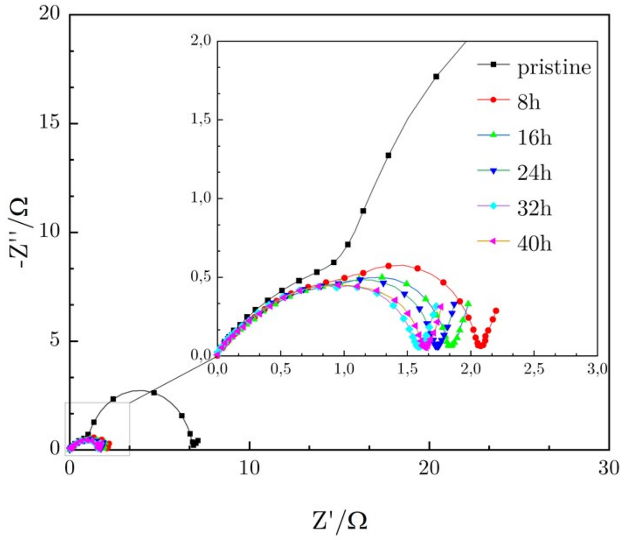

Figure S3. EIS in the negative half-cell.

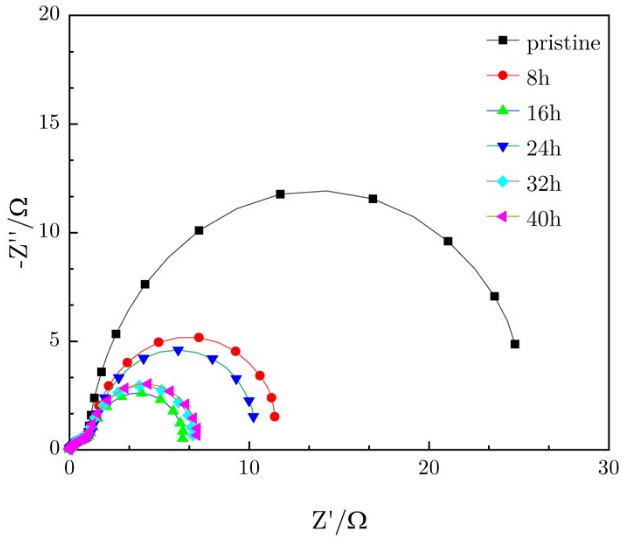

Figure S4. EIS in the positive half-cell.

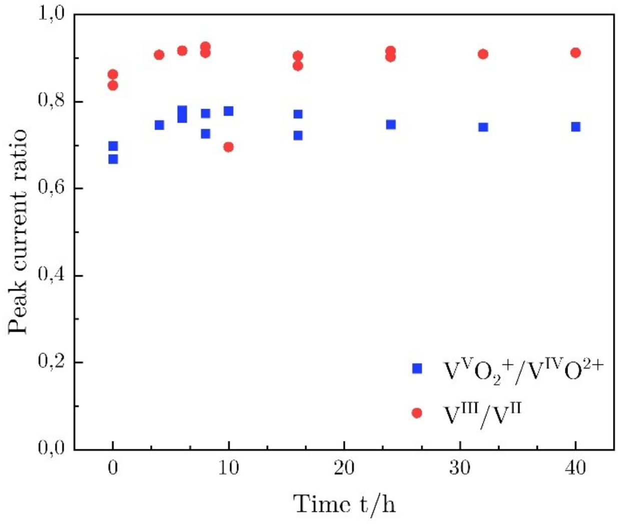

Figure S5. Peak current ratios with regard to the thermal activation time.

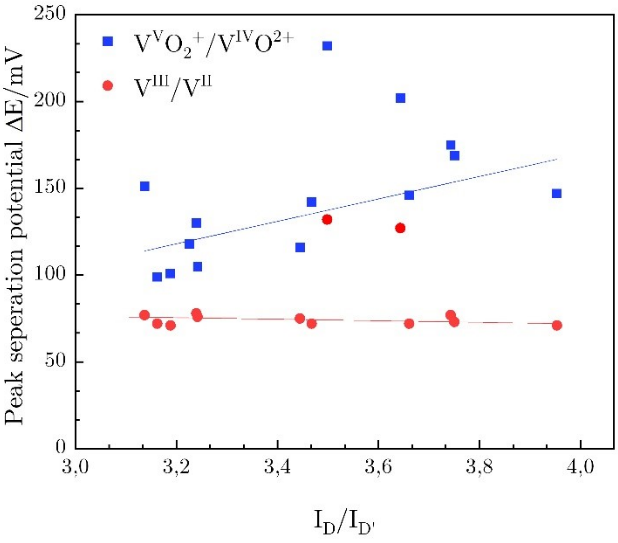

Figure S6. Peak separation potential *vs*. *I*(D)/*I*(D’) ratio.

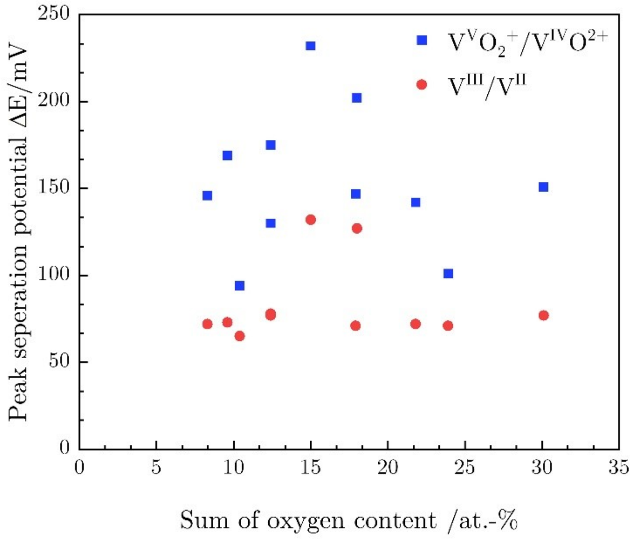

Figure S7. Peak potential separation *vs*. oxygen concentration.
